# Peer effect of enterprise innovation: Empirical evidence from China

**DOI:** 10.3389/fpsyg.2022.921127

**Published:** 2022-10-25

**Authors:** Li Liu, Jiguo Yang, Minna Zheng, Linlin Jin

**Affiliations:** ^1^School of Management, Henan University of Technology, Zhengzhou, China; ^2^School of Marxism, Henan University of Technology, Zhengzhou, China; ^3^School of Economics and Management, Hebei University of Technology, Tianjin, China; ^4^Business School, Zhengzhou University of Aeronautics, Zhengzhou, China

**Keywords:** peer effect, enterprise innovation, managerial ability, enterprise size, economic policy uncertainty

## Abstract

Innovation investment is crucial to enterprise development and economic growth. As peer enterprises face similar market environment and development prospects, they pay attention to the innovation activities of peer enterprises in the industry because of economic rationality or the idea of seeking advantages and avoiding disadvantages. This paper aims to investigate the interaction and channel of enterprise innovation behavior of peer effect based on the data of Chinese share-listed enterprises from 2010 to 2021. The results show that peer effect exists in the innovation behavior of enterprises. We also provide evidence that managerial ability is the mechanism of the peer effect of enterprise innovation. In addition, we find that small-scale enterprises are more likely to be affected by the innovation behavior of peer enterprises compared with large enterprises. More importantly, we reveal that economic policy uncertainty significantly negatively regulates the peer effect of enterprise innovation.

**JEL classification:**

G30, G31, O31

## Introduction

Innovation is the key factor to promote economic development and an important means to maintain the company’s competitive advantage (Kim and Koo, 2018). The realization of the national innovation development strategy and upgrading of economic structure depend on continuous R&D investment and the improvement of innovation ability (Wang et al., 2019). Enterprise innovation ability is one of the important factors that affect the company value and business performance. Positive innovation strategies can provide a continuous driving force for the healthy and sustainable development of enterprises. In 2021, there are 298 thousand enterprises with valid invention patents in China, an increase of 52 thousand over the previous year, and the enterprise has 1.908 million effective invention patents, with a year-on-year increase of 22.6%. What drives an enterprise innovative and what makes enterprises more involved in innovation investment have attracted the attention of more and more academic researchers over the last decades. It is increasingly important for policymakers and academic researchers to master the determinants of R&D investment driven by enterprises, as it is the basis of various R&D issues (Peng et al., 2020).

Innovation is the result of enterprises’ ability to absorb and apply both internal and external knowledge to business purposes (Wang and Chung, 2013). In a highly international environment, Chinese enterprises can compete effectively only when their innovation ability is better than main competitors in the international market (Wang and Chung, 2020). Scholars have proved theoretically and empirically that innovation has a positive impact on enterprise performance (Peng and Tao, 2022), export of enterprise (Chen S. et al., 2022), enterprise value (Hao et al., 2022), structure upgrading (Ye et al., 2020), and economic development (Bilgin et al., 2021). In addition, existing literature has studied the influencing factors of innovation at all levels, such as the characteristics of managers (Chen X. H. et al., 2022), enterprise level (Xia et al., 2022), and inter-enterprise level (Woods et al., 2022). Most empirical studies on enterprise innovation are based on the assumption that R&D investment choices are often made independently of peer enterprise behavior or affected by enterprise-specific determinants (Leary and Roberts, 2014). However, previous studies show that peer enterprises have frequent competition and interaction, and the similar market and institutional environment make it have the basic conditions to imitate peer behavior. Therefore, in the decision-making process, enterprises not only consider their own factors, but also pay attention to similar decisions of enterprises with similar status. Enterprises choose to follow other enterprises with similar characteristics to avoid risks such as uneconomical cost and uncertain results caused by individual ability and resource constraints. That is, their innovation decision-making is greatly affected by the external environment (Joo et al., 2016; Mai and Lin, 2021). Therefore, the R&D investment policy choice of enterprises is affected by the behavior of peer enterprises (Xue and Zhao, 2021).

This phenomenon is called peer effect (Manski, 2000). The research on peer effect originated from sociology and gradually expanded to the fields of economics and management. Peer effect refers to the interaction between individuals in the same group, and the behavior and results of an individual are affected by their peer behavior and results (Gyimah et al., 2020). People’s behavior is affected by consciousness (Smith et al., 2012; Habib et al., 2021), but their behavior is also social (Göckeritz et al., 2010) because of the social relationship (Blay et al., 2018), and their decision-making will be affected by other people in the group (Yin et al., 2021). Therefore, there is an active interaction between the decision-maker and peers in behavioral decision-making. The spillover effect of peer behavior causes the fluctuation of decision-makers’ behavior at the reference group level to be several times that at the individual level (Zhong and Zhang, 2018). It can be seen that the main source of peer effect lies in the limited rationality of managers and the uncertainty of decision-making results. Peer effect breaks the relevant assumptions of independent decision-making and believes that enterprises in the same group face a similar living environment. They have the conditions and motivation to compete or imitate learning, which makes enterprises consciously pay attention to the behavior of peer enterprises. In this way, enterprises can avoid the costs and risks of independent decision-making (Gortner and Weele, 2019; Gyimah et al., 2020), obtain more information related to decision-making, and maintain their competitive advantage (Lieberman and Asaba, 2006).

Relevant studies have found that there is an obvious peer effect in enterprise decision-making. Lieberman and Asaba (2006) explain business imitation behavior from information theory and competition theory. From the perspective of information theory, incomplete information is the main reason for enterprise imitation. Enterprises will follow other peer enterprises with superior information. From the perspective of competition theory, enterprises imitate the decisions of other enterprises to maintain a relative position in the market. According to Chen and Ma (2017), peer effects affect enterprises’ investment decisions if enterprises are faced with fierce competition from peer groups and higher quality of information disclosure. Leary and Roberts (2014) conclude that peer effect is important than other factors that affect the decision of corporate capital structure. The impact of this clustering effect also exists in other important decisions of the enterprise, such as capital structure (Fairhurst and Nam, 2018), cash holding (Qiu and Wan, 2015; Chen et al., 2019), corporate investment (Frésard and Valta, 2016; Bustamante and Frésard, 2021), debt maturity structure (Duong et al., 2015), stock split line (Kaustia and Rantala, 2015), and dividend policy (Grennan, 2019; Yan and Zhu, 2020). However, how this peer effect affects the innovation decision-making of enterprises has not received enough attention. Few studies take the innovation behavior of peer enterprises as an important factor affecting the competitiveness of enterprises to study the interaction between them, and the enterprise innovation mechanism is still in a “black box” state. Few of the existing studies take enterprise innovation as an important factor affecting enterprises and study the interaction between them. In addition, the innovation effect of peer enterprises is often ignored in the existing empirical research. Therefore, we study whether the innovation decisions of peer enterprises can have an impact on the innovation behavior of a single enterprise.

The innovation behavior of enterprises has strong sociality, which makes the innovation achievements have strong spillover effect (Park et al., 2020). Previous studies have shown that innovation investment can create positive externalities in the form of innovation and technology spillovers (Sun et al., 2021). Therefore, technological knowledge spillovers can reduce R&D costs and encourage other enterprises to increase innovation investment (Lin et al., 2021). Previous empirical studies on enterprise innovation have assumed that innovation decisions are made independently within the enterprise, which ignore the contribution of external factors that play an important role in the competitive market (Turner et al., 2010). Therefore, it is necessary for enterprises to formulate innovation strategies to keep up with the development of the industry, which contain all available information about the innovation activities of their peers. In addition, enterprises pay attention to the R&D behavior of industry competitors and adjust their R&D decisions accordingly to maintain market competitiveness. It is worth noting that although the literature has studied the determinants of enterprise innovation from many aspects (Xue et al., 2021), the impact of peer innovation behavior has not been thoroughly discussed.

Our study speaks to three strands of existing literature. First, this paper provides a new idea for studying enterprise innovation behavior from the perspective of peer effect. Previous studies have reported evidence of peer effects on capital structure, cash holding, corporate investment, debt maturity structure, stock split line, and dividend policy. Unlike Chen S. et al. (2022), who focus on the impact of external innovation of stakeholders such as upstream enterprises, downstream enterprises, and competitors on enterprise exports, we extend the peer effect to enterprise innovation, because imitation is irreversible and has a high degree of information asymmetry, which requires a lot of capital investment over very long periods, resulting in higher imitation costs. It is important to focus on the impact of peers on enterprise innovation, because industry dynamics or strategic interaction can amplify the positive and negative impacts unique to enterprises within and between industries, which is particularly important in the field of innovation. Given that enterprise innovation is increasingly becoming the main driver of economic growth, it is crucial to understand how innovation dynamics in the industry affect peer enterprises. We extend peer effect to the field of enterprise innovation and focus on whether there is peer effect in enterprise innovation behavior. More particularly, we further analyze whether this peer effect is different in enterprises of different sizes.

Second, we contribute to the existing literature by providing new evidence about the mechanism of peer effect of enterprise innovation. Although the previous literature confirms that enterprise innovation is affected by other enterprise in the industry (Brown et al., 2009; Bui et al., 2021), it is not clear through which channel peer effect of enterprise innovation. The inherent uncertainty of innovation activities makes managers’ evaluation of innovation investment crucial. Managers may hesitate to adopt innovative strategies when uncertainty is high. As a reflection of managers’ handling of complex problems and decision-making behavior, managerial ability may be an important channel for enterprises to innovate companion enterprises. Therefore, we further explore the mechanism of managerial ability in the peer effect of enterprise innovation. By testing the role of managerial ability, we find that peer enterprises affect the innovation decisions of other enterprises in the industry through managerial ability.

Our third contribution is to expand the research on innovation by studying the impact of economic policy uncertainty on the peer effect of enterprise innovation. Although previous studies mostly discussed the influencing factors of enterprise innovation investment behavior from the perspective of internal factors or external macro environment (Yang and Yang, 2010; Sung, 2019; He and Wang, 2020), these studies cannot determine whether the peer decision-making of innovation investment is different when enterprises are faced with different degrees of uncertainty in the economic policy environment. Therefore, we bring the macroeconomic environment into the analysis framework and further study the regulatory effect of economic policy uncertainty on the peer effect of enterprise innovation. This study highlights a new influencing factor of innovation, which can enrich our understanding of peer effect and enterprise innovation decision-making.

The remainder of this paper is the following. Section “Theoretical background and research hypothesis” introduces theoretical background and hypothesis development. Section “Empirical design” describes data sources and sample selection, definition of variable, and model design. Section “Empirical results” provides empirical results, including descriptive statistics, analysis of regression results, and robustness tests. Section “Further analysis” presents further analysis, including heterogeneity of enterprise size and moderating effect of economic policy uncertainty. Section “Conclusion and implications” concludes the paper and some policy implications.

## Theoretical background and research hypothesis

### Peer effect of enterprise innovation

Information asymmetry theory holds that there are differences in the information obtained by individuals in economic activities, and the amount of information has an impact on future decision-making. According to the uncertainty reduction theory, individuals identify with some groups because they feel uncertainty. They reduce or control the uncertainty they feel by identifying with others (Hogg, 2007). Considering the cost and uncertainty of obtaining information, enterprises may refer to the decisions of other enterprises in the same industry or with similar attributes, which is called peer effect (An et al., 2016). Therefore, enterprises pay close attention to the behavior of industry competitors and adjust their relevant decisions accordingly to maintain market competitiveness (Mark et al., 2014). The competitive relationship between peers enables them to have frequent and strong interaction, which makes the behavior decision-making between organizations stimulating and radiating. Meanwhile, the highly competitive market environment leads to an increase of bankruptcy risk. At this time, the management strategy of peer enterprises helps to reduce the risk of decision-making failure, which makes enterprises have a higher enthusiasm to follow suit and imitate peer enterprises (Chen and Ma, 2017). When behavior is uncertain, such as making innovation decisions, individual behavior is significantly affected by other individuals in the group and enterprises will imitate the innovation decisions of enterprises with similar characteristics. Therefore, imitation is an important way to promote innovation diffusion. The theory of technological imitation represented by Mansfield holds that the diffusion of technological innovation can be realized through the imitation of innovation (Mansfield, 1985). Additionally, innovation is crucial to the long-term development of enterprises. Innovation has a significant impact on the future production and operation of enterprises and even changes the industrial competition pattern.

Enterprises in the same industry face similar market environment and development prospects. They not only need to seize the market and form defense barriers through innovation competition, but also need innovation to resist market risks and achieve consumption leadership (Leary and Roberts, 2014). On the one hand, enterprise innovation is a kind of exploratory behavior, which leads to strong uncertainty in decision-making and behavior results. However, peer enterprises face the same industry and market environment. The complexity of innovation investment decision-making and the uncertainty of results urge enterprises to refer to the corresponding behavior of similar groups to reduce uncertainty. On the other hand, innovation is an important strategy of an enterprise. The innovation level of enterprises has a significant impact on their future production and operation and even changes the competitive pattern of the industry. Therefore, enterprises pay close attention to the innovation decisions of peer enterprises and respond positively to maintain its competitive position. Compared with enterprises with good performance, enterprises with poor performance are more motivated to get out of trouble through innovation strategy. This means that the performance of an enterprise can be used as the boundary condition for imitating the innovation behavior of its learning peers. In addition, innovation activities are highly specialized. Whether the business is similar is an important standard for enterprises to choose imitation learning objects (Dierynck and Verriest, 2020). Consequently, the business differences between enterprises affect the degree of imitation and learning from the innovation behavior of peer enterprises. However, it is difficult for enterprises to obtain all the information about the innovation behavior of peer enterprises in time. Therefore, enterprises should always pay attention to the innovation decisions of peer enterprises (Sharapov and Ross, 2019). Enterprise can reduce the cost of searching information by imitating the R&D activities of peer enterprises (Marvin and Lieberman, 2006). This can not only maintain the existing market position of enterprises, but also avoid risks to maintain core competitiveness. Therefore, the innovation decision-making of enterprises is affected by the innovation behavior of peer enterprises. Based on the above analysis, this paper puts forward the following hypothesis:

Hypothesis 1. There is peer effect in enterprise innovation behavior.

### The mediating effect of managerial ability

Neoclassical economic theory believes that managers are homogeneous and can be completely replaced. The choices made by the enterprise are exactly the same if the external environment is the same. However, there are differences in the decision-making behavior of enterprises in the real market, and this difference cannot be explained by the factors of company characteristics and industry characteristics (Cheng and Wang, 2019). According to the differences of enterprise management and the bounded rationality of people, Hanbrick and Mason put forward the Upper Echelons Theory in 1984. This theory believes that the characteristics of management differences will affect enterprise decision-making and then affect enterprise performance. The managerial ability not only reflects the knowledge, experience, and cognition of managers, but also reflects the managerial ability to deal with complex problems and make decisions. Therefore, the managerial ability is the comprehensive embodiment of the diversity of managers, which inevitably affects the realization of enterprise innovation decision-making (Zhang, 2021) and performance goals (Duan, 2021). Previous studies have shown that the managerial ability significantly affects the correctness of decision-making (Hambrick, 2007; Demerjian et al., 2013). In particular, the complex and changeable industry and market environment makes the innovation investment decision-making of enterprises have certain risks. Therefore, managerial ability plays an important role in the innovation decision-making process of enterprises.

The long cycle and high uncertainty of innovation investment mean that enterprises need sufficient market information support when making R&D decisions. The complex information in the market increases the cost of searching information for enterprises, resulting in managers’ excessive reliance on decision-making information in the industry (Sushil et al., 1998). Managers with high ability can timely capture market changes. They integrate the internal and external resources of the enterprise and make reasonable R&D decisions to promote the development of the enterprise when the market information is scarce. With these individual advantages, these managers reduce the imitation behavior of enterprises in the process of innovation investment (Peter et al., 2012). Enterprises with information advantages are in an active position and grasp the resources with potential economic value, which make them better predict the market development direction and make investment decisions by taking advantage of market opportunities. Under the leadership of competent managers, the information advantage of enterprises has been strengthened, which makes it easier for enterprises to seize innovative investment opportunities and grow into leaders with higher positions in the industry. The R&D behavior of these industry-leading enterprises has attracted the attention of industry followers, resulting in more imitation behavior.

In addition, the highly competitive market environment increases the uncertainty of enterprise management. The management strategy of peer enterprises helps to reduce the risk of decision failure, which makes enterprises have a strong enthusiasm to imitate peer enterprises (Chen et al., 2019). Managers with high ability have rich experience in corporate governance, so that they can be keenly aware of the information contained in the changes in the market environment, identify the potential risks in innovative investment projects, and adjust innovation strategies to avoid the failure of innovative investment. In addition, these competent managers can find various potential factors in the company’s resources that promote the success of innovation investment activities in the process of innovation, which can improve the level of enterprise innovation. Meanwhile, enterprise managers attach great importance to maintaining their own reputation, and enterprises lacking innovative spirit will be abandoned by the public or even eliminated by the market. Therefore, enterprise managers have the motivation to make innovation investment that is not lower than the average level of peer enterprises, which can avoid damage to their own reputation and establish a good social image. Against this background, this paper puts forward the following hypothesis:

Hypothesis 2. The managerial ability is an important mechanism of enterprise innovation peer effect.

## Empirical design

### Data sources and sample selection

China’s R&D investment data have been disclosed since 2009. Therefore, we use the data of China’s A-share-listed enterprises from 2010 to 2021 as the research sample. The final sample is obtained by screening this sample with the following conditions: (1) financial and insurance listed companies are excluded; (2) listed companies with relevant data are excluded; (3) ST companies are removed; (4) industries with less than two enterprises in the same year are eliminated; (5) major events and business changes that have occurred in the data range are deleted. The final sample observation value is 6,888. In addition, all continuous variables are winsorized at the level of 1 and 99% to avoid the interference of outliers. The data required for the study are mainly from China Stock Market and Accounting Research Database and Wind Database. Some missing data can be found and supplemented through the official websites of Shanghai Stock Exchange, Shenzhen Stock Exchange, listed companies, and Sina Finance website.

### Definition of variable

R&D innovation needs a long process with cycle and uncertain results. Some innovation inputs may not be capitalized and eventually transformed into intangible assets. Based on the research of Liu and Jiang, R&D expenditure/total operating revenue is used to measure enterprise innovation (Liu and Jiang, 2016).

Following Leary and Roberts (2014), we regard enterprises in the same industry as peers and take the average value of innovation of other enterprises in the same industry as the proxy variable of innovation level of peer enterprises. This measurement method avoids the endogenous problem of the model, highlights the cross interactive relationship between enterprises in the same industry, and more accurately tests the peer effect of enterprise green technology innovation.

Referring to the method of Demerjian et al. (2012) and Demerjian et al. (2013), we use data envelopment analysis (DEA) and Tobit model to measure the managerial ability. First, DEA is used to calculate the total efficiency of enterprise operation (*Score*). Among them, the output variable is the enterprise’s operating revenue (*Sales*), and the input variable includes net value of fixed assets (*Ppe*), net value of intangible assets (*Intan*), R&D expenses (*R&D*), operating cost (*Cost*), sum of sales and management expenses (*Sae*), and net goodwill (*Gw*). The calculation is as shown in Eq. 1,


$$M\,a\,x\,\_\,S\,c\,o\,r\,e_{t}=$$

(1) $$\frac{S\,a\,l\,e\,s_{t}}{\varphi_{1}\,P\,P\,e_{t}+\varphi_{2}\,I\,n\,\tan_{t}+\varphi_{3}\,R\&D_{t}+\varphi_{4}\,C\,o\,s\,t_{t}+\varphi_{5}\,S\,a\,e_{t}+\varphi_{6}\,G\,w_{t}}$$

Second, the managerial ability is estimated, because the *Scor*e calculated by DEA analysis is affected by both enterprise factors and manager factors. Therefore, we establish model (2) to control the influencing factors at the year and enterprise level, and the residual after regression ε_*t*_ is the managerial ability.


$$\text{Tobit}\,\left(S\,c\,o\,r\,e_{t}\right)=\alpha_{0}+\alpha_{1}\,S\,i\,z\,e_{t}+\alpha_{2}\,F\,c\,f_{t}+\alpha_{3}\,M\,s_{t}+\alpha_{4}\,F\,c\,l_{t}$$

(2) $$+\alpha_{5}\,A\,g\,e_{t}+\alpha_{6}\,D\,i\,v_{t}+\sum Y\,e\,a\,r+\sum I\,n\,d\,u\,s\,t\,r\,y+\varepsilon_{t}$$

*Size* is the natural logarithm of the total assets of the enterprise. *Fcf* is the enterprise free cash flow level. If the enterprise free cash flow is positive, the index value is 1; otherwise, it is 0. *Ms* is the market share, which is measured by the proportion of enterprise operating revenue in industry operating revenue. *Fcl* refers to the degree of internationalization, which is measured by the proportion of overseas sale revenue in operating revenue. *Age* is the natural logarithm of the year of establishment of the enterprise. *Div* is the business complexity of the enterprise, which is measured by the sum of the square of the income of each business department divided by the total income of the enterprise.

Referring to the existing literature (Zhang, 2015; Guney et al., 2017), we control some variables that affect enterprise innovation. These control variables include return on total assets (*Roa*), tangible asset ratio (*Tang*), cash asset ratio (*Cash*), enterprise age (*Age*), asset-liability ratio (*Lev*), and Tobin *Q* value (*Tobin’Q*). The measurement method of corresponding variables of peer enterprises is consistent with that of peer enterprises. The variables and their definitions in this paper are shown in Table 1.

**TABLE 1:** Definition of variables.

Name	Symbol	Definition
R&D	*Rd*	R&D expenditure/total operating income
R&D in the same industry	*Mrd*	Average R&D of other enterprises in the same industry
Managerial ability	*Ma*	Referring to the method of Demerjian et al. (2012) and Demerjian et al. (2013)
Return on total assets	*Roa*	Net profit/total assets
Tangible asset ratio	*Tang*	Total tangible assets/total assets
Cash asset ratio	*Cash*	Cash assets/total assets
Enterprise age	*Age*	Years of establishment
Asset liability ratio	*Lev*	Total liabilities/total assets
Tobin’Q	*Tobin’Q*	Market value/net assets
Return on total assets in the same industry	*Mroa*	Average value of *Roa* of peer enterprises
Tangible asset ratio in the same industry	*Mtang*	Average value of *Tang* of peer enterprises
Cash asset ratio in the same industry	*Mcash*	Average value of *Cash* of peer enterprises
Enterprise age in the same industry	*Mage*	Average value of *Age* of peer enterprises
Asset liability ratio in the same industry	*Mlev*	Average value of *Lev* of peer enterprises
Tobin’Q in the same industry	*Mtobin’Q*	Average value of *Tobin’Q* of peer enterprises
Industry	*Ind*	Dummy variable
Year	*Year*	Dummy variable

### Model design

To examine Hypothesis 1, we estimate the model (3):


(3) $$R\,d_{i,t}=\alpha +\alpha_{1}\,M\,r\,d_{i,t}+\alpha \,C\,o\,n\,t\,r\,o\,l\,s_{i,t}+I\,n\,d\,u\,s\,t\,r\,y_{i}+Y\,e\,a\,r_{t}+\varepsilon_{i,t}$$

To investigate the mediating effect of managerial ability, we construct the following model:


(4) $$M\,a_{i,t}=\alpha +\alpha_{1}\,M\,r\,d_{i,t}+\alpha \,C\,o\,n\,t\,r\,o\,l\,s_{i,t}+I\,n\,d\,u\,s\,t\,r\,y_{i}+Y\,e\,a\,r_{t}+\varepsilon_{i,t}$$

$$R\,d_{i,t}=\alpha +\alpha_{1}\,M\,r\,d_{i,t}+\alpha_{2}\,M\,a_{i,t}+\alpha \,C\,o\,n\,t\,r\,o\,l\,s_{i,t}+I\,n\,d\,u\,s\,t\,r\,y_{i}$$

(5) $$+Y\,e\,a\,r_{t}+\varepsilon_{i,t}$$

where the indices _*i*_ and _*t*_ denote enterprise and year, respectively. The dependent variable _*Rd_it_*_ is the innovation level of enterprise _*i*_ in year _*t*_. The independent variable _*Mrd_it_*_ is peer enterprise’s innovation level. The mediating variable _*Ma_it_*_ represents the transmission path of enterprise innovation peer effect. _*Control_it_*_ is a set of control variables. _*Industry_it_*_ and _*Year_it_*_ represent the fixed effect of industry and year, respectively, and _ε_it__ is the error term. In Eq. 3, the coefficient _α_1__ represents the peer effect of enterprise innovation. In Eqs 4, 5, _α_1__ and _α_2__ denote mediating effect of managerial ability.

## Empirical results

### Descriptive statistics

Table 2 presents the descriptive statistics of variables in this paper. The average of *Rd* is 0.043, the maximum value is 0.075, and the minimum value is 0, which shows that R&D investment scale of Chinese enterprises is not high. The maximum value of *Ma* is 0.040 and the minimum value is 0, which indicates that there are great differences in enterprises. The values of the control variables are shown in Table 2. We also calculate the variance inflation factors (VIF) of the variables to ensure unbiased regression results. It is found that the VIF value of all variables is less than 3, suggesting that multicollinearity is not a serious problem in this paper.

**TABLE 2:** Descriptive statistics.

	Mean	S.D.	Max	Min
*Rd*	0.043	0.052	0.075	0.000
*Mrd*	0.040	0.037	0.168	0.000
*Ma*	−0.028	1.112	0.209	–0.373
*Roa*	0.037	0.052	0.195	–0.159
*Tang*	0.956	0.067	1.000	0.612
*Cash*	0.152	0.121	0.595	0.008
*Age*	17.139	5.278	27.000	9.000
*Lev*	0.433	0.218	0.872	0.051
*Tobin’Q*	2.190	1.815	9.967	0.000
*Mroa*	0.040	0.028	0.088	–0.031
*Mtang*	0.928	0.034	0.982	0.786
*Mcash*	0.158	0.049	0.350	0.076
*Mage*	17.202	1.573	21.387	14.429
*Mlev*	0.428	0.087	0.631	0.239
*Mtobin’Q*	2.176	0.715	4.875	0.932

This table lists the mean, standard deviation (S.D.), maximum (Max), and minimum (Min) values of variables in this paper.

### Analysis of regression results

#### Peer effect of enterprise innovation

The peer effect of enterprise innovation is shown in Table 3. Column (1) shows that the regression coefficients of *Mrd* are 0.224 and significant at the 1% level. A 1 SD increase in innovation investment of peer enterprises leads to 22.4 % point increase in the innovation investment of other enterprises in the industry. This result means that there is peer effect in enterprise innovation behavior, that is, innovation activities have spillover effects. Other enterprises in the industry increase innovation investment when peer enterprises carry out innovation activities. Therefore, Hypothesis 1 is supported. In addition, *Roa*, *Cash*, *Mroa*, and *Mlev* have a significantly positive effect on *Rd*, whereas *Tang*, *Age*, *Mtang*, and *Mcash* have a significantly negative effect on *Rd*. In addition, the coefficients of *Lev*, *TobinQ*, *Mage*, and *MtobinQ* are not significant at the significance level. The results of the control variables are consistent with the existing literature (Hang et al., 2016; Jiang and Zhang, 2018; Mo et al., 2020).

**TABLE 3:** Regression results.

Variable	*Rd*	*Ma*	*Rd*
	(1)	(2)	(3)
*Mrd*	0.224*** (4.036)	0.158* (1.727)	0.234*** (4.229)
*Ma*			0.183** (2.310)
*Roa*	0.013*** (3.559)	0.008* (1.701)	0.026*** (3.343)
*Tang*	−0.018* (−1.759)	0.002 (1.014)	−0.020* (−1.773)
*Cash*	1.220* (1.887)	1.315 (1.208)	1.359* (1.821)
*Age*	−0.582*** (−2.837)	−0.746** (−2.125)	−0.438** (−2.004)
*Lev*	0.120 (0.024)	0.143 (0.127)	0.162 (0.031)
*Tobin’Q*	−0.002 (−0.724)	0.005 (0.536)	−0.001 (−0.683)
*Mroa*	0.164* (1.855)	0.037 (1.008)	0.125* (1.723)
*Mtang*	−1.112** (−2.347)	0.083 (1.001)	−1.228* (−1.724)
*Mcash*	−0.121* (−1.837)	0.097 (0.903)	−0.129 (−1.603)
*Mage*	−0.546*** (−3.714)	−0.630* (−1.690)	−0.425** (−2.338)
*Mlev*	0.373 (0.278)	0.204 (0.128)	0.371 (0.283)
*Mtobin’Q*	−0.010 (−0.353)	0.001 (0.582)	−0.012 (−0.435)
*Year*	Yes	Yes	Yes
*Indu*	Yes	Yes	Yes
*Adjusted R-squared*	0.187	0.201	0.192

***, **, and * indicate significant at the level of 1, 5, and 10%, respectively. T statistics are enclosed in parentheses.

#### The mediating effect of managerial ability

Columns (2) and (3) of Table 3 report the mediating effect of managerial ability in the peer effect of enterprise innovation. Column (2) shows that the regression coefficients of *Mrd* is 0.158 and significant at the 10% level, which suggests that a 1% increase in innovation investment of peer enterprises, and the managerial ability will increase by 0.158. This means that there is a positive correlation between the innovation activities of peer enterprises and managerial ability. Column (3) shows that the regression coefficients of *Mrd* are 0.234 and significant at the 1% level, and regression coefficients of *Ma* are 0.183 and significant at the 5% level. It can also be seen from the regression results that enterprise innovation behavior affects the innovation decision-making of peer enterprises through the managerial ability. In the context of information asymmetry, the stronger the ability of managers, the more conducive to give play to the advantages of searching information and reduce the degree of information asymmetry between enterprises and the market. Therefore, Hypothesis 2 is supported.

### Robustness test

#### Alternative measures of enterprise innovation

Referring to the research of He and Wintoki (2016) and Xu and Zhao (2019), we use the ratio of R&D expenditure to operating revenue to measure enterprise innovation. The results of peer effect of enterprise innovation are shown in column (1) of Table 4. The coefficient of *Mrd* is 0.314 and significant at the 1% level, which is consistent with the above research conclusion. This shows that the peer effect of enterprise innovation is robust. The results of the mediating effect of managerial ability are shown in column (2) and column (3) of Table 4. The coefficient of *Mrd* and *Ma* is significant, demonstrating that the regression results are robust.

**TABLE 4:** Robustness test.

Variable	*Rd*1	*Ma*	*Rd*1	*Mrd*	*Rd*
	(1)	(2)	(3)	(4)	(5)
*Mrd*	0.314*** (4.537)	0.128* (1.782)	0.327*** (4.662)		0.236*** (4.115)
*Ma*			0.206** (2.332)		
*Malpha*				0.125* (1.714)	
*Roa*	0.025** (2.402)	0.004 (1.616)	0.021** (2.330)	0.016** (2.013)	0.008** (2.159)
*Tang*	−0.013* (−1.751)	0.001 (1.230)	−0.026* (−1.849)	−0.020* (−1.738)	−0.037* (−1.782)
*Cash*	1.342** (2.450)	1.001 (1.075)	1.286* (1.725)	1.237** (2.339)	1.536* (1.839)
*Age*	−0.552*** (−3.016)	−0.479*** (−2.864)	−0.463*** (−2.945)	−0.455*** (3.187)	−0.508*** (−2.997)
*Lev*	0.153 (0.057)	0.107 (0.139)	1.008 (0.071)	1.231 (0.083)	0.105 (0.079)
*Tobin’Q*	−0.001 (−0.787)	0.010 (0.349)	−0.005 (−0.886)	−0.007 (−0.758)	−0.022 (−0.680)
*Mroa*	0.197* (1.836)	0.015 (0.902)	0.210* (1.873)	0.118* (1.828)	0.155* (1.773)
*Mtang*	−0.997*** (2.756)	0.065 (1.233)	−1.317* (−1.895)	−1.039* (−1.743)	−1.208* (−1.826)
*Mcash*	−0.287** (−2.334)	0.020 (0.820)	−0.315** (−2.001)	−0.336** (−2.164)	−0.106* (−1.728)
*Mage*	−0.305** (−2.300)	−0.528** (−2.057)	−0.473** (−2.132)	−0.552** (−2.006)	−0.336*** (−3.983)
*Mlev*	0.264 (0.227)	0.353 (0.158)	0.371 (0.225)	0.289 (0.341)	0.389 (0.205)
*Mtobin’Q*	−0.008 (−0.336)	0.001 (0.523)	−0.007 (−0.250)	−0.009 (−0.397)	−0.017 (−0.405)
*Year*	Yes	Yes	Yes	Yes	Yes
*Indu*	Yes	Yes	Yes	Yes	Yes
*Adjusted R-squared*	0.210	0.223	0.225	0.189	0.203

***, **, and * indicate significant at the level of 1, 5, and 10%, respectively. T statistics are enclosed in parentheses.

#### Endogenesis

Based on the research of Leary and Roberts (2014), we choose stock return alpha as the instrumental variable of enterprise innovation to avoid endogenous problems. The information of enterprise’s innovation investment is reflected in the change of stock price, especially after excluding external factors such as market and industry, the change information of enterprise’s own stock price can be presented more accurately (Sood and Tellis, 2009). It can be seen that the stock returns of peer enterprises are only related to the innovation level of peer enterprises, but not to the innovation level of a certain enterprise. Therefore, we take the average stock return alpha (*Malpha*) of peer enterprises as the instrumental variable of peer enterprise innovation (*Mrd*).

Columns (4) and (5) in Table 4 report the results of instrumental variable regression. The test results of weak instrumental variables show that the *F* value is 103.227, which is greater than 10 and significant at the level of 1%, indicating that the selected instrumental variables have a strong correlation with the innovation of peer enterprises. It can be seen from column (2) that the coefficient of *Malpha* is 0.022 and significant at the 1% level. This result means that the average alpha of peer enterprises is positively correlated with the average innovation of peer enterprises. The results of column (3) can be found the coefficient of *Mrd* is 0.326 and significant at the 1% level, which shows that the average innovation level of peer enterprises improves the R&D intensity of enterprises.

## Further analysis

### Heterogeneity of enterprise size

Enterprise size is an important factor affecting the level of innovation investment. Enterprises in different sizes have different influences and responses to innovation investment of peer enterprises. First, enterprises in different sizes have adopted different innovation investment strategies. Large enterprises have the advantages of abundant funds and mature management, which makes them tend to carry out exploratory research to achieve industry-leading breakthrough innovation. Compared with large enterprises, small enterprises are unable to afford scientific research projects with large costs. However, small enterprises have more flexibility than large enterprises, which makes them tend to incremental innovation (Cockburn and Hederson, 2001; Koberg et al., 2003). Second, because large enterprises have huge social networks, they can obtain more information in the process of contacting upstream and downstream enterprises. This information is helpful to the generation of innovative investment ideas and the formulation of innovative investment strategies (Kim et al., 2009). Due to the limited social network of small enterprises, they cannot grasp the information on the market in time. It may be that only after large enterprises have carried out innovation investment, they can obtain relevant information and follow large enterprises in innovation investment.

Therefore, large enterprises not only have the ability to bear the high risks and large expenses brought by R&D, but also have more information on innovation investment. This makes large enterprises more inclined to carry out breakthrough innovation projects that require large-scale investment, which is conducive to the output of innovation achievements. However, small enterprises have insufficient R&D resources, weak access to information, and insufficient funds, leading them to follow their peer enterprises in innovation investment. It can be seen that enterprise size plays a stronger exemplary role in peer enterprises. Following Sung (2019), we use the total assets of enterprises to measure the enterprise size. According to the regulations on the classification standards for small- and medium-sized enterprises issued by the Ministry of Industry and Information Technology of China in 2011, we divide enterprises into two groups. Enterprises with less than 1,000 employees or operating income of less than 400 million Yuan are classified as small- and medium-sized enterprises, and others are large enterprises. The estimation results are in Table 5. The coefficient of *Mrd* is not significant in subsample of large enterprise, while the regression coefficient of *Mrd* is 0.462 and it is significant at the 1% level in susbample of small enterprise. It can be seen that the innovation behavior of smaller enterprises is more significantly affected by peer enterprises. Compared with large enterprises, small enterprises have less innovation investment information and investment scale, which makes it easier for them to follow the innovation decisions of their peer enterprises.

**TABLE 5:** Subsample grouping regression results with different enterprise sizes.

Variable	Large enterprise		Small enterprise	
	Coefficient	*t*	Coefficient	*t*
*Mrd*	0.257	1.258	0.462***	3.067
*Roa*	0.020**	2.269	0.038***	3.698
*Tang*	−0.019*	–1.887	−0.012**	–2.061
*Cash*	1.306*	1.829	1.429*	1.863
*Age*	−0.552**	–2.063	−0.616**	–2.668
*Lev*	0.130	0.012	0.121	0.057
*Tobin’Q*	–0.003	–0.682	–0.005	–0.091
*Mroa*	0.128*	1.773	0.203*	1.895
*Mtang*	−1.139**	–2.209	−1.220**	–2.671
*Mcash*	−0.206*	–1.782	−0.268*	–1.808
*Mage*	−0.338**	–2.287	−0.439***	–2.652
*Mlev*	0.297	0.435	0.304	0.550
*Mtobin’Q*	–0.009	–0.442	–0.008	–0.429
*Ind*	Yes	Yes	Yes	Yes
*Year*	Yes	Yes	Yes	Yes

***, **, and * indicate significant at the level of 1, 5, and 10%, respectively.

### Moderating effect of economic policy uncertainty

All activities of enterprises are carried out in the environment, and the important feature of the environment is uncertainty. China’s economy is in a period of transformation from high-speed growth to high-quality development, which makes enterprises in a highly uncertain environment and increases the risk of enterprise innovation activities (Cheung et al., 2010). Enterprise innovation is an investment activity with high risk, large investment, and long-term characteristics. Therefore, enterprise innovation activities are vulnerable to changes in the market environment, and economic policy uncertainty is an important incentive for the changing market environment (Francis et al., 2014). Economic uncertainty refers to the uncertainty caused by the inability of enterprises to reasonably predict and accurately evaluate the expected changes in the economic system and the future distribution of economic results (Jurado et al., 2016).

Economic policy uncertainty may affect the innovation activities of peer enterprises through the acquisition and transmission of information. Enterprise innovation activities have stronger uncertainty when the degree of economic policy uncertainty is relatively high (Ghosh and Olsen, 2009). At this time, the problem of market information asymmetry is more serious, which limits the same group effect under competitive motivation. The improvement of market information asymmetry increases the difficulty of enterprise management in predicting the future economic policy situation and strengthens the perception of external risks (Gulen and Ion, 2016). Therefore, enterprises feel pessimistic because it is difficult to predict future earnings and risks and then reduce or even give up innovation investment to hedge risks.

It takes time for enterprises to obtain innovation-related information from their peer enterprise. Enterprises are habitually rigid when facing threats from the external market environment (Soh, 2009). The higher the uncertainty of the economic policy, the higher the search cost and difficulty of this search activity, because high economic uncertainty means that it is difficult for enterprises to grasp the external economic policy environment. At this time, the cost and risk of technology transfer between enterprises are relatively large, but the efficiency of technology transfer is relatively low, which is not conducive to learning and communication between enterprises (Zeng et al., 2020). Therefore, the higher the degree of economic policy uncertainty, the greater the restrictions on enterprises’ access to resources and information through peer enterprise R&D signals, which inhibits the enterprise’s innovation momentum. At the same time, the high uncertainty of economic policy hinders the information transmission of innovation activities among peer enterprises. The peer effect of enterprise R&D investment will be weakened when the information flow between enterprises is blocked.

Therefore, economic policy uncertainty has an inhibitory effect on the peer effect of enterprise innovation. Drawing on the research of Baker et al. (2016), we use the macroeconomic policy uncertainty index jointly released by Stanford University and the University of Chicago to measure China’s economic policy uncertainty (*Epu*). The index is based on the South China Morning Post in Hong Kong, China, and is widely used in research on policy uncertainty. Since the economic uncertainty index is monthly data, we use the geometric average of monthly data within a year to process this index as an annual measurement index to better match the sample data. The regression results are shown in Table 6. The coefficient of *Epu* is −0.035, which is significant at the 10% level, and the coefficient of *Mrd* × *Epu* is −0.156, which is significant at the 1% level. This finding shows that with the increase of economic policy uncertainty, enterprise innovation is less affected by the same group of enterprises. Therefore, economic policy uncertainty negatively regulates the peer effect of enterprise innovation.

**TABLE 6:** Moderating effect of economic policy uncertainty.

Variable	*Rd*	
	Coefficient	*t*
*Mrd*	0.551***	3.996
*Epu*	−0.035**	–2.338
*Mrd* × *Epu*	−0.156***	–7.672
*Roa*	0.017***	4.016
*Tang*	−0.008**	–2.143
*Cash*	1.703*	1.829
*Age*	−0.517*	–1.776
*Lev*	0.380	0.062
*Tobin’Q*	–0.017	–0.175
*Mroa*	0.534**	2.230
*Mtang*	−1.817*	–1.758
*Mcash*	−0.339**	–2.302
*Mage*	−0.671**	–2.157
*Mlev*	0.446	0.683
*Mtobin’Q*	–0.004	–0.550
*Ind*	Yes	Yes
*Year*	Yes	Yes

***, **, and * indicate significant at the level of 1, 5, and 10%, respectively.

## Conclusion and implications

### Conclusion

According to the empirical results, the conclusion of this paper are as follows: (1) Peer effect exists in the innovation behavior of enterprises, and the innovation behavior of enterprises in the same industry can drive each other. (2) Managerial ability plays an important mediating effect in the peer effect of enterprise innovation. The information advantage of enterprises has been strengthened under the leadership of competent managers, which makes it easier for enterprises to seize innovative investment opportunities and grow into leaders with higher positions in the industry. The R&D behavior of these industry-leading enterprises has attracted the attention of industry followers, resulting in more imitation behavior. (3) There are differences in the impact of enterprises of different sizes on the innovation investment behavior of peer enterprises. Compared with large enterprises, the innovation behavior of smaller enterprises is more significantly affected by peer enterprises. (4) Economic policy uncertainty significantly negatively regulates the peer effect of enterprise innovation, that is, economic policy uncertainty weakens the convergence of innovation among enterprises. The peer effect of enterprise innovation is more significant when the economic policy is relatively stable. The peer effect of enterprise innovation will be weakened when economic policies fluctuate violently.

### Policy implications

The implications of this paper are as follows:

(1) Our empirical results show that the peer effect of innovation behavior enables innovation activities to spread among enterprises. It is suggested that policymakers adopt peer learning mechanism to guide and encourage enterprise innovation. We should use the peer effect to promote enterprise innovation and turn passive innovation activities into voluntary behaviors of enterprises. Peer effect can promote enterprises’ active innovation, in which the innovation vitality of market players has been stimulated and their creativity has been continuously enhanced. Government departments can promote the exchange of innovation information among peer enterprises by creating a group innovation atmosphere to promote the level of regional innovation.
(2) We confirm the mediating effect of managerial ability in the peer effect of enterprise innovation. Therefore, it is necessary to give full play to the mediating effect of enterprise managers in the process of using peer effect to promote enterprise innovation. Enterprises need to cultivate managers’ awareness of innovation and make managers realize the importance of innovation to the long-term development of enterprises. Meanwhile, enterprises should encourage managers to use social networks to timely understand the innovation information of other enterprises and actively learn from the innovation investment experience of peer enterprises.
(3) We find that there are differences in the response of enterprises of different sizes to the innovation behavior of peer enterprises. The Chinese government can set up innovation benchmarks in various industries, improve the innovation level of the whole industry and society through these benchmark enterprises, and focus the incentive on those benchmark enterprises with exemplary role to improve the effect of government promoting enterprise innovation investment.
(4) The Chinese government needs to reasonably control the frequency of economic policy adjustment to maintain the relative robustness of economic policy, which can reduce the negative impact of economic policy uncertainty on enterprise innovation peer effect. Relevant departments should strive to build a good external economic environment to help enterprises give better play to their innovation vitality. For example, relevant departments should pay attention to the role of government subsidies and increase support for enterprises with innovation potential, which can improve the operating conditions of enterprises and better promote enterprise innovation.

## Data availability statement

Publicly available datasets were analyzed in this study. These data can be found here: https://www.wind.com.cn/portal/en/Home/index.html and https://www.gtarsc.com/.

## Author contributions

LL formulated the conceptual framework, designed the model, analyzed the data, and wrote the manuscript. JY formulated the conceptual framework, designed the model, obtained inference, and wrote the manuscript. MZ and LJ analyzed the data and provided editorial supports. All authors contributed to the article and approved the submitted version.
